# Integration of SFTSV Viral Load, Age, and Double‐Negative B‐Cells as Prognostic Biomarkers for Severe Fever With Thrombocytopenia Syndrome Outcomes

**DOI:** 10.1155/jimr/8554086

**Published:** 2026-01-15

**Authors:** Wei Wei, Qian Tai, Heng Liu, Rujia Chen, Yun Wang, Ting Wang, Renren Ouyang, Shiji Wu, Feng Wang, Hongyan Hou

**Affiliations:** ^1^ Department of Laboratory Medicine, Tongji Hospital, Tongji Medical College, Huazhong University of Science and Technology, Wuhan, Hubei, China, hust.edu.cn

**Keywords:** age, B-cell, double negative B-cells, prognosis, severe fever with thrombocytopenia syndrome, SFTSV

## Abstract

**Background:**

Severe fever with thrombocytopenia syndrome (SFTS) is a tick‐borne viral illness with high mortality, yet early risk stratification remains challenging. Recent evidence suggests that B‐cell dysregulation contributes to disease severity.

**Methods:**

A cohort of 168 patients with confirmed SFTS was retrospectively analyzed. Flow cytometry was used to quantify B‐cell subsets in peripheral blood at admission. Laboratory markers, viral load, and B‐cell phenotypes were evaluated for prognostic relevance using univariate and multivariate Cox regression analyses. Receiver operating characteristic (ROC) curves and nomogram models were employed to assess predictive value.

**Results:**

Deceased patients exhibited significantly higher viral loads, elevated proinflammatory cytokines (interleukin‐6 (IL‐6), IL‐10, and tumor necrosis factor‐alpha (TNF‐α)), and markers of multiorgan dysfunction. Immunophenotyping revealed a reduction in naïve B‐cells (IgD^+^CD27^−^), alongside expansion of double negative B‐cells (DNBs) (IgD^−^CD27^−^) in fatal cases. Furthermore, viral load was positively correlated with inflammatory cytokines and dysfunctional B‐cell subsets, suggesting that impaired humoral immunity contributes to persistent hyperinflammation in severe SFTS. Multivariate Cox regression analysis identified higher viral load (HR = 2.193, *p*  < 0.001), older age (HR = 1.073, *p*  < 0.001), and increased proportion of DNBs (HR = 1.024, *p* = 0.035) as independent predictors of 28‐day mortality. A combined prognostic model integrating these variables achieved excellent performance (AUC = 0.906, 95% CI: 0.814–0.967), significantly surpassing individual markers and enabling early identification of high‐risk SFTS patients.

**Conclusion:**

This study demonstrates that integrating B‐cell subset profiling with laboratory indicators significantly improves early prognostic assessment in SFTS. These findings provide insights into immune‐pathological mechanisms and support timely risk stratification and intervention to reduce mortality.

## 1. Introduction

Severe fever with thrombocytopenia syndrome (SFTS) is an emerging acute infectious disease caused by *Bandavirus dabieense* (formerly known as SFTS virus, SFTSV), a tick‐borne virus belonging to class *Bunyavicetes* [1]. Over the past decades, the geographic distribution of SFTS has expanded, with cases reported across 27 provinces in China and an increasing incidence annually [2]. This disease has also been documented in other Asian countries, including South Korea, Japan, and Vietnam [3–5]. SFTSV is primarily transmitted through tick bites, but human‐to‐human transmission via blood contact or aerosol exposure has also been reported [6–8]. The typical clinical characteristics of SFTS include fever, thrombocytopenia, leukopenia, gastrointestinal symptoms, and, in severe cases, progress to multiorgan failure [9]. With a case fatality rate ranging from 5% to 30%, SFTS poses a growing public health threat and has been listed as a priority pathogen by the World Health Organization [9].

Dysregulation of host immune response has been widely implicated in the pathogenesis of severe SFTSV infection [10, 11]. Fatal cases of SFTS are often characterized by impaired virus‐specific humoral immunity [12]. The delayed or absent neutralizing antibody production leads to ineffective viral clearance, persistent inflammatory activation, and eventual multiorgan damage [13]. Postmortem analyses have revealed infiltration of activated B‐cells and plasmablasts in secondary lymphoid organs, with some cells harboring SFTSV antigens [14]. During the acute phase of infection, transient yet extensive expansion of plasmablasts is commonly observed in the peripheral blood of SFTSV infection [15, 16]. Single‐cell RNA sequencing (scRNA‐seq) of peripheral blood mononuclear cells (PBMCs) has further demonstrated that a hyperactive type I interferon (IFN‐I) response correlates with heightened inflammation and unfavorable prognosis [17]. Moreover, in vitro studies suggest that SFTSV‐infected B‐cells can secrete proinflammatory cytokines such as interleukin‐6 (IL‐6) and tumor necrosis factor‐alpha (TNF‐α), driving plasmablast differentiation and potentially contributing to immune dysregulation [15]. These findings underscore the central role of B‐cell dysfunction and humoral immune failure in the progression and poor outcomes of SFTS.

Excessive inflammatory responses play a central role in the pathogenesis of SFTSV infection. Elevated levels of cytokines such as IL‐6, IL‐10, TNF‐α, and interferon‐gamma (IFN‐γ) are frequently observed in severe or fatal cases, contributing to cytokine storm, vascular leakage, and multiorgan failure [18, 19]. These inflammatory markers have been closely associated with poor clinical outcomes in SFTS patients. In addition, a range of laboratory indicators, including high viral load, prolonged coagulation times (e.g., APTT, PT), thrombocytopenia, and elevated markers of hepatic and renal dysfunction such as lactate dehydrogenase (LDH), alanine aminotransferase (ALT), and blood urea nitrogen (BUN), have also been linked to increased disease severity and mortality. These findings highlight the multifactorial nature of SFTS progression, which involves both immune‐mediated damage and direct viral cytopathology. However, most current prognostic models rely solely on general laboratory parameters and lack integration with detailed immune profiling. Given the emerging evidence of disrupted B‐cell‐mediated humoral immunity in fatal cases, a combined analysis of laboratory markers and B‐cell subset dynamics may improve early risk stratification and offer novel insights into SFTSV pathogenesis.

This study aimed to identify prognostic indicators in SFTS by integrating laboratory markers with B‐cell subset profiling. By combining clinical and immunological parameters, we sought to improve early risk stratification and provide novel insights to guide timely and targeted clinical interventions, thereby reducing disease‐related mortality.

## 2. Patients and Methods

### 2.1. Patients

This retrospective study included 168 patients diagnosed with SFTS at Tongji Hospital from March 2022 to June 2024. The diagnosis of SFTS was confirmed by the detection of SFTSV RNA in serum using real‐time polymerase chain reaction (PCR), combined with characteristic clinical features such as severe fever, thrombocytopenia, and hemorrhagic symptoms. Patients were stratified into survival and deceased groups based on their 28‐day clinical outcomes from symptom onset. Written informed consent was obtained from all participants, and the study received approval from the ethical committee of Tongji Hospital, Tongji Medical College, Huazhong University of Science and Technology (TJ‐IRB20230632).

### 2.2. Clinical Data Collection

Clinical data collection: The clinical data were retrieved from the electronic medical records of Tongji Hospital and included demographic information, comorbidities (e.g., hypertension, cardiovascular disease, diabetes, tuberculosis, and hepatitis B), clinical symptoms, treatment measures, and 28‐day outcomes. All peripheral blood samples for laboratory testing were collected at hospital admission. Routine laboratory tests were performed within 24 h of admission. Laboratory parameters encompassed hematologic indices (leukocytes, lymphocytes, hemoglobin, and platelets), liver and renal function markers (ALT, AST, GGT, LDH, total protein, albumin, urea, creatinine, uric acid, and eGFR), inflammatory markers (ferritin, PCT, and hsCRP), cytokines (IL‐1β, IL‐2R, IL‐6, IL‐8, IL‐10, and TNF‐α), and coagulation indices (APTT, PT, TT, D‐dimer, and fibrinogen) [20].

### 2.3. Flow Cytometry for BCell Subpopulations

Heparinized peripheral blood samples were collected from patients upon admission. A total of 100 μL of whole blood was incubated with a predefined monoclonal antibody panel for B‐cell immunophenotyping. The following antibodies were used: anti‐CD38‐FITC (BD Pharmingen, HB7), anti‐CD19‐PE/Cy7 (SJ25C1), anti‐CD27‐PerCP (2D1), CD45‐V500C (2D1), and anti‐IgD‐APC (IA6‐2). After 15 min of incubation at room temperature, red blood cells were lysed using a commercial lysing buffer. The remaining leukocytes were washed, resuspended in 300 μL phosphate‐buffered saline (PBS), and analyzed on a BD FACS Lyric flow cytometer. A representative gating strategy for B‐cell subpopulations is shown in Figure S1.

### 2.4. Statistical Analysis

Continuous variables were compared using Student’s *t*‐test or Mann–Whitney *U* test, and categorical variables using the chi‐square test. Receiver operating characteristic (ROC) curve analysis was performed to determine optimal cut‐off values and assess predictive performance. Pearson correlation was used for association analysis. Prognostic factors were identified using univariable and multivariable Cox proportional hazards regression, with significant variables from univariable analysis included in the multivariable model. Statistical analyses were conducted using GraphPad Prism 9.5 and SPSS 22.0, with *p*  < 0.05 considered statistically significant.

## 3. Results

### 3.1. Demographics and Clinical Characteristics of SFTS Patients

Among 168 confirmed SFTS patients, 97 (57.7%) survived and 71 (42.3%) died within 28 days. The overall cohort included 89 males (53.0%) and 79 females (47.0%), with no significant difference in sex distribution between the survival and deceased groups (*p* = 0.435). The deceased group was significantly older than the survivors (mean age: 67.2 vs, 61.4 years, *p*  < 0.001). Consciousness disorder was notably more prevalent in non‐survivors (45.1% vs, 15.5%, *p* < 0.001). Diabetes and hemophagocytosis were more common among deceased patients (*p* = 0.019 and *p* < 0.001, respectively). The median time from symptom onset to admission was 7 days (IQR 5–8), with no significant difference between survival and deceased groups (*p* = 0.152). Regarding treatment, deceased patients more frequently required continuous renal replacement therapy (CRRT) (39.4% vs, 13.4%, *p* < 0.001) and mechanical ventilation (25.4% vs 1.0%, *p* < 0.001), reflecting more severe disease progression (Table 1).

**Table 1:** Demographics and clinical characteristics of SFTS patients on admission.

Parameters	Total (*n* = 168)	Survival (*n* = 97)	Deceased (*n* = 71)	*p* Value
Age, mean (SD) year	63.869 (9.386)	61.423 (9.528)	67.211 (8.126)	<0.001
Sex	—	—	—	0.269
Male, n (%)	71 (42.262)	37 (38.144)	34 (47.887)	—
FeMale, n (%)	97 (57.738)	60 (61.856)	37 (52.113)	—
Symptoms, n (%)				
Fever	159 (94.643)	95 (97.938)	64 (90.141)	0.037
Fatigue	51 (30.357)	27 (27.835)	24 (33.803)	0.509
Myalgia	14 (8.333)	10 (10.309)	4 (5.634)	0.423
Inappetence	33 (19.643)	19 (19.588)	14 (19.718)	1.000
Nausea	45 (26.786)	29 (29.897)	16 (22.535)	0.375
Vomiting	53 (31.548)	30 (30.928)	23 (32.394)	0.973
Abdominal pain	17 (10.119)	13 (13.402)	4 (5.634)	0.164
Diarrhea	73 (43.452)	43 (44.330)	30 (42.254)	0.912
Headache and dizziness	41 (24.405)	22 (22.680)	19 (26.761)	0.67
Altered consciousness disorder	47 (27.976)	15 (15.464)	32 (45.070)	**<0.001**
Comorbidities, n (%)				
Hypertension	53 (31.548)	26 (26.804)	27 (38.028)	0.168
Diabetes	13 (7.738)	3 (3.093)	10 (14.085)	**0.019**
Coronary disease	19 (11.310)	10 (10.309)	9 (12.676)	0.817
Hemophagocytosis	81 (48.214)	30 (30.928)	51 (71.831)	**<0.001**
Days from symptom onset to admission, median (IQR)	7 (5, 8)	7 (5, 8)	6 (5, 8)	0.152
Therapy, n (%)				
Corticosteroid	56 (33.333)	29 (29.897)	27 (38.028)	0.348
CRRT	41 (24.405)	13 (13.402)	28 (39.437)	**<0.001**
Mechanical ventilation	19 (11.310)	1 (1.031)	18 (25.352)	**<0.001**

*Note*: The differences of the parameters were compared between the survival and deceased group. *p* values in bold indicate statistical significance (*p* < 0.05).

Abbreviations: CRRT, continuous renal replacement therapy; SD, standard deviation.

### 3.2. Laboratory Parameters Between Survival and Deceased Groups of SFTS Patients

Significant laboratory differences were observed between survival and deceased patients with SFTS (Table 2). Platelet counts were significantly decreased in the deceased group (*p* = 0.002). Liver function markers, including ALT, AST, and LDH, as well as renal function indicators such as urea, creatinine, and uric acid, were significantly elevated (*p* < 0.01). Coagulation profiles demonstrated prolonged TT, PT, and APTT, accompanied by elevated D‐dimer levels in fatal cases. Inflammatory and cytokine markers, including ferritin, PCT, hsCRP, IL‐6, IL‐8, IL‐10, and TNF‐α, were also markedly increased in the deceased group. Notably, TGF‐β levels were also significantly lower in deceased patients compared with survivors (*p* = 0.019), reflecting impaired immunoregulatory cytokine responses in fatal SFTS. The SFTSV RNA viral load was also significantly higher among the deceased group (median log_10_: 4.835 vs., 3.117, *p* < 0.001).

**Table 2:** Comparison of laboratory markers between the survival and deceased groups of patients with SFTS.

Parameters	Total (*n* = 168)	Survival (*n* = 97)	Deceased (*n* = 71)	*p* Value
Hematological marker				
WBC (× 10^9^/L)	3.855 (2.612;5.475)	3.760 (2.530;5.580)	4.220 (2.695;5.445)	0.512
Neutrophiles (× 10^9^/L)	73.500 (56.275;83.725)	72.200 (53.500;83.100)	74.300 (59.600;84.450)	0.371
Lymphocytes (× 10^9^/L)	19.400 (12.325;34.025)	19.400 (11.700;34.900)	19.100 (12.550;29.650)	0.494
Monocytes (× 10^9^/L)	4.900 (2.600;8.625)	5.900 (3.300;9.200)	4.000 (2.300;7.450)	**0.014**
RBC (× 10^12/^L)	4.236 (0.732)	4.218 (0.670)	4.265 (0.826)	0.739
Hemoglobin (g/L)	338.492 (10.107)	338.438 (10.329)	338.574 (9.862)	0.942
Platelet (× 10^9^/L)	45.500 (31.750;63.250)	50.000 (36.000;70.000)	38.000 (27.000;54.000)	**0.002**
Liver function marker				
ALT (U/L)	84.500 (46.000;163.500)	75.000 (42.000;132.500)	105.000 (61.500;205.500)	**0.017**
GGT (U/L)	39.000 (25.000;87.750)	35.000 (22.000;64.000)	46.000 (30.000;131.500)	**0.007**
AST (U/L)	165.000 (76.500;356.250)	127.000 (73.000;282.500)	243.000 (96.500;525.500)	**0.003**
LDH (U/L)	776.500 (469.500;1352.000)	631.000 (390.000;924.500)	1191.000 (701.500;1723.000)	**<0.001**
ALP (U/L)	72.500 (59.250;103.000)	68.000 (54.500;90.000)	84.000 (66.500;141.500)	**<0.001**
Total protein (g/L)	60.603 (6.065)	61.659 (6.224)	59.190 (5.580)	**0.008**
Albumin (g/L)	32.428 (4.582)	33.317 (4.483)	31.223 (4.468)	**0.003**
Globulin (g/L)	27.650 (25.400;30.000)	28.200 (25.550;30.300)	27.600 (25.200;29.650)	0.591
TBIL (μmol/L)	8.100 (5.825;11.375)	7.500 (5.650;10.750)	8.900 (6.050;12.450)	0.106
DBIL (μmol/L)	4.300 (2.725;6.600)	3.800 (2.600;5.500)	5.000 (3.350;8.100)	**0.007**
IBIL (μmol/L)	3.200 (2.000;4.975)	3.500 (2.100;5.050)	2.800 (1.900;4.650)	0.186
Amylopsin (U/L)	83.000 (52.000;139.000)	80.000 (50.000;129.500)	96.000 (55.000;175.000)	0.106
Lipase (IU/L)	172.700 (97.100;345.800)	153.650 (90.175;262.200)	211.300 (122.500;552.600)	**0.004**
Triglyceride (mmol/L)	1.980 (1.350;2.710)	2.030 (1.355;2.715)	1.875 (1.368;2.645)	0.78
Total cholesterol (mmol/L)	3.045 (2.525;3.552)	3.150 (2.700;3.635)	2.790 (2.455;3.375)	**0.027**
Kidney function marker				
Urea (mmol/L)	5.985 (4.425;8.700)	5.210 (3.900;6.800)	7.100 (5.300;12.000)	**<0.001**
Uric acid (μmol/L)	247.000 (193.000;338.000)	243.500 (182.250;283.500)	287.000 (218.900;403.000)	**0.001**
Creatinine (μmol/L)	85.000 (67.250;115.500)	77.000 (63.500;100.500)	98.000 (75.500;136.500)	**<0.001**
Coagulation marker				
TT (S)	26.100 (21.500;38.700)	23.000 (20.600;28.900)	32.550 (25.850;54.475)	**<0.001**
PT (S)	12.900 (12.300;13.700)	12.800 (12.200;13.500)	13.300 (12.600;14.600)	**0.001**
APTT (S)	57.750 (44.900;69.250)	52.650 (41.450;61.950)	64.000 (54.250;85.650)	**<0.001**
Fibrinogen (g/L)	2.580 (2.230;3.010)	2.730 (2.370;3.115)	2.355 (2.105;2.825)	**0.002**
D‐dimer (μg/mL FEU)	2.980 (1.620;6.635)	2.190 (1.220;4.040)	5.530 (3.095;9.990)	**<0.001**
Inflammatory marker				
hsCRP (mg/L)	4.500 (1.650;12.700)	2.800 (1.350;7.800)	7.050 (2.775;19.500)	**0.002**
Ferritin (μg/L)	10562.000 (3985.075;22495.750)	5719.550 (1853.000;13337.750)	19903.500 (8823.575;41753.750)	**<0.001**
PCT (ng/mL)	0.330 (0.120;0.900)	0.185 (0.090;0.472)	0.630 (0.300;1.750)	**<0.001**
Cytokine marker				
IL‐1β (pg/mL)	5.300 (5.000;11.600)	5.000 (5.000;10.550)	6.950 (5.000;15.950)	**0.02**
IL‐2R (U/mL)	1257.500 (907.000;1829.750)	1068.500 (754.250;1403.750)	1811.000 (1177.250;2334.750)	**<0.001**
IL‐6 (pg/mL)	41.000 (15.638;118.800)	28.980 (10.010;55.115)	110.200 (44.720;334.700)	**<0.001**
IL‐8 (pg/mL)	29.200 (16.900;84.400)	23.100 (13.600;34.950)	52.100 (28.875;128.750)	**<0.001**
IL‐10 (pg/mL)	60.000 (20.300;147.000)	28.700 (15.250;87.350)	127.000 (61.650;205.250)	**<0.001**
TNF‐α (pg/mL)	25.350 (16.375;46.200)	19.350 (14.300;33.450)	38.850 (23.400;84.025)	**<0.001**
TGF‐β (pg/mL)	4.507 (3.111;8.944)	5.136 (3.663;76.190)	3.663 (2.462;7.131)	**0.019**
Log_10_ (SFTSV_RNA)	3.712 (2.535;4.735)	3.117 (2.199;3.968)	4.835 (3.754;5.608)	**<0.001**

*Note*: The data were shown as median and interquartile range (IQR). *p* values in bold indicate statistical significance (*p* < 0.05).

Abbreviations: ALP, alkaline phosphatase; ALT, alanine aminotransferase; APTT, activated partial thromboplastin time; AST, aspartate aminotransferase; DBIL, direct bilirubin; eGFR, estimated glomerular filtration rate; GGT, glutamyltransferase; hsCRP, high‐sensitivity C‐reactive protein; IBIL, indirect bilirubin; IL, interleukin; LDH, lactate dehydrogenase; PCT, procalcitonin; PT, prothrombin time; RBC, red blood cell; TBIL, total bilirubin; TNF‐α, tumor necrosis factor alpha; TT, thrombin time; WBC, white blood cell.

### 3.3. B Cell Subpopulation Analysis Among SFTS Patients

The proportion of naïve B‐cells (IgD^+^CD27^−^) was significantly reduced in the deceased group, whereas double negative B‐cells (DNBs) (IgD^−^CD27^−^) were markedly increased (Figure 1a,b). Plasmablasts (CD27^+^CD38^high^) showed robust expansion during the acute phase of SFTSV infection; however, their levels did not differ significantly between survivors and deceased patients. Similarly, no significant differences were observed in the proportions of unswitched memory B‐cells (IgD^+^CD27^+^) and switched memory B‐cells (IgD^−^CD27^+^) (Figure 1b). These findings suggest that fatal SFTS is associated with a skewed B‐cell profile, characterized by a reduction in naïve B‐cells and an accumulation of DNBs.

Figure 1Alterations in B cell subsets among SFTS patients. (a) Representative flow cytometry plots of B cell subsets in the survival and deceased groups. CD19^+^ B cells were first gated, followed by identification of plasmablasts as CD27^+^CD38^high^ cells. Non‐plasmablast B cells were further classified into naïve (IgD^+^CD27^-^), unswitched memory (IgD^+^CD27^+^), switched memory (IgD^-^CD27^+^), and double‐negative (DN, IgD^−^CD27^−^) subsets based on IgD and CD27 expression. (b) Comparison of B cell subset proportions between survival and deceased groups.  ^∗∗^ *p* < 0.01,  ^∗∗∗^ *p* < 0.001.

**Figure:**
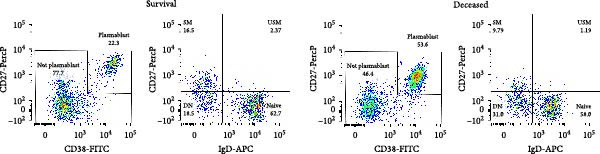
(a)

**Figure:**
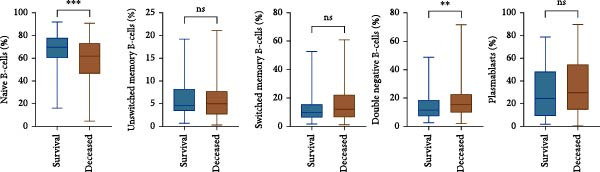
(b)

### 3.4. Association Between Viral Load, BCell Subsets, and Inflammatory Cytokines

Correlation analyses demonstrated that higher SFTSV RNA viral loads were positively associated with increased levels of IL‐6 (*R* ^2^ = 0.226, *p*  < 0.001), TNF‐α (*R* ^2^ = 0.192, *p*  < 0.001), and IL‐10 (*R* ^2^ = 0.286, *p*  < 0.001) (Figure 2a). In addition, viral load was significantly correlated with elevated proportions of total B‐cells (*R* ^2^ = 0.147), DNBs (*R* ^2^ = 0.158), and plasmablasts (*R* ^2^ = 0.136) (Figure 2b). Notably, total B‐cell percentages were also positively correlated with IL‐6 (*R* ^2^ = 0.0923), TNF‐α (*R* ^2^ = 0.0896), and IL‐10 (*R* ^2^ = 0.0664) levels (all *p*  < 0.01) (Figure 2c), suggesting that B‐cell activation may contribute to the heightened inflammatory response observed in severe SFTSV infection.

Figure 2Correlations among viral load, B cell subsets, and inflammatory cytokines in SFTS patients. (a) Positive correlations between Log_10_ (SFTSV_RNA) and inflammatory cytokines IL‐6, TNF‐α, and IL‐10. (b) Higher viral loads were associated with increased proportions of total B cells, double negative B cells, and plasmablasts. (c) Total B cell percentages were positively correlated with IL‐6, TNF‐α, and IL‐10 levels.

**Figure:**
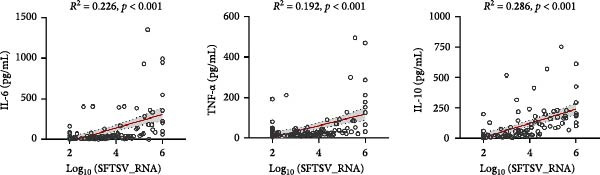
(a)

**Figure:**
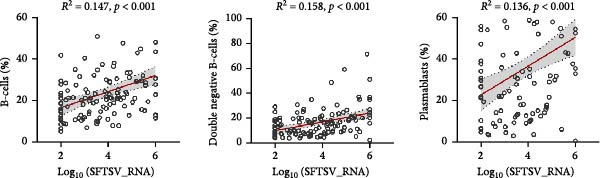
(b)

**Figure:**
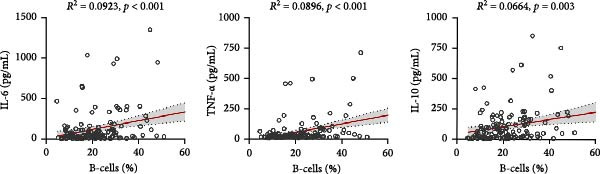
(c)

### 3.5. Cox Regression Analyses for Prognostic Prediction of Fatal SFTSV Infection

**Figure 4:**
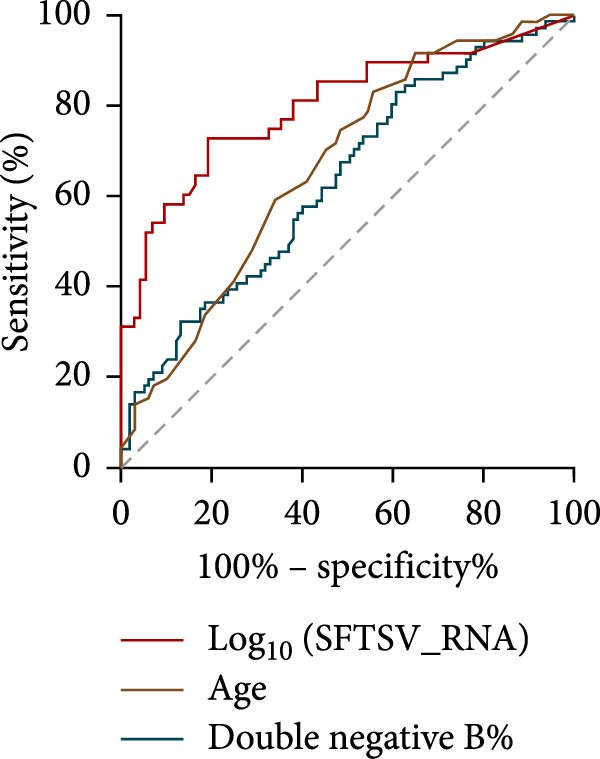
ROC curves for individual prognostic indicators.

To identify independent predictors of fatal outcomes in patients with SFTS, multivariate Cox proportional hazards regression was performed. Higher SFTSV RNA viral load (HR = 2.193; 95% CI: 1.649–2.917; *p*  < 0.001), older age (HR = 1.073; 95% CI: 1.034–1.113; *p*  < 0.001), and elevated percentage of DNBs (HR = 1.024; 95% CI: 1.002–1.048; *p* = 0.035) were independently associated with 28‐day mortality (Figure 3a). The combined model demonstrated excellent discrimination with a 28‐day AUC of 0.906 (95% CI: 0.814–0.967) (Figure 3b). Furthermore, a prognostic nomogram integrating viral load, age, and DNB percentage was constructed to estimate individualized survival probability (Figure 3c). The predictive performance of individual indicators was also evaluated. SFTSV RNA viral load showed the best discrimination (AUC = 0.802), followed by age (AUC = 0.669) and DNB percentage (AUC = 0.635), with viral load also exhibiting the highest Youden index (0.54) and accuracy (0.607) (Table 3, Figure 4). These results highlight the superior prognostic utility of the combined model over individual indicators for predicting outcomes in patients with SFTS.

Figure 3Prognostic evaluation model for 28‐day mortality in SFTS patients. (a) Receiver operating characteristic (ROC) curve showing the discriminative ability of the combined model incorporating age, SFTSV RNA viral load, and double negative B cell percentage. (b) Forest plot of multivariate Cox proportional hazards regression analysis identifying Log_10_ (SFTSV_RNA), age, and double negative B cells as independent predictors of 28‐day mortality. (c) Nomogram based on the multivariable Cox model, integrating viral load, age, and double negative B cell proportion to estimate individualized 28‐day survival probability.

**Figure:**
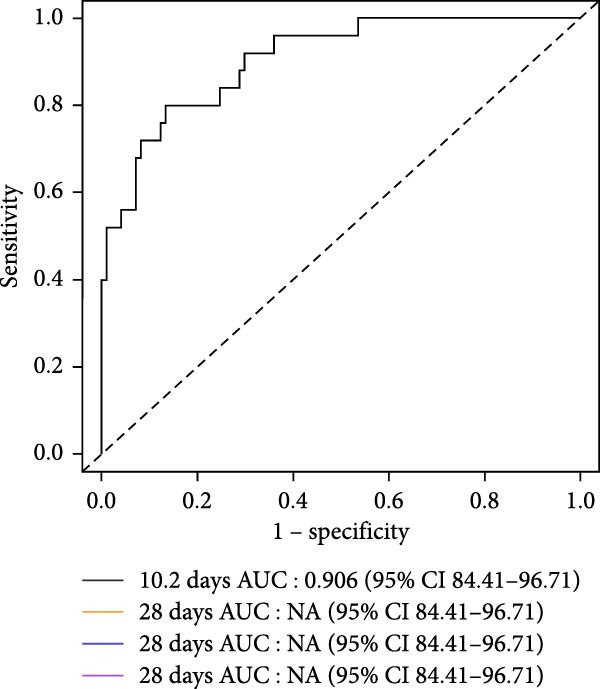
(a)

**Figure:**
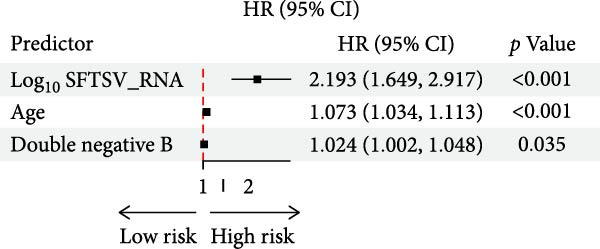
(b)

**Figure:**
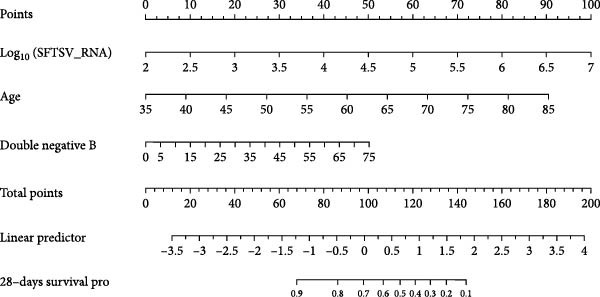
(c)

**Table 3:** Diagnostic performance of individual prognostic indicators for predicting 28‐day mortality in SFTS patients.

Parameters	AUC	Sensitivity	Specificity	Youden index	Cutoff	Accuracy
Log10SFTSV_RNA)	0.802	0.729	0.811	0.54	4.076	0.607
Age	0.669	0.831	0.443	0.274	59	0.577
Double negative B%	0.635	0.831	0.392	0.223	8.95	0.577

## 4. Discussion

SFTS remains a significant public health concern due to its high mortality rate and limited therapeutic options. The nonspecific clinical presentation underscores the importance of comprehensive laboratory evaluation to guide prognosis and intervention. In our cohort, a detailed comparison between survival and deceased patients revealed substantial systemic derangements in those with fatal outcomes.

Macrophages are major producers of IL‐6 and IL‐10 during viral infections, and SFTSV can directly infect monocytes/macrophages, inducing excessive cytokine release [21]. Elevated levels of proinflammatory cytokines, particularly IL‐6, IL‐10, and TNF‐α, were prominently observed in the deceased group, consistent with the cytokine storm phenotype known to drive multiorgan dysfunction and mortality in severe viral infections [22–24]. The immunosuppressive cytokine IL‐10 plays a critical role in immune paralysis during severe infections. SFTSV nonstructural proteins are known to induce IL‐10 production, while miR‐146 b‐mediated M2 macrophage polarization further enhances viral replication and immune evasion [13, 25, 26]. Our findings align with these mechanisms: deceased patients exhibited not only markedly elevated IL‐10 levels but also higher viral loads and widespread immune dysfunction, suggesting a feed‐forward loop of immunosuppression and viral persistence. The significant correlations between viral load, IL‐10, and aberrant B‐cell subsets in our study further support this interplay between inflammatory and immunosuppressive pathways in the pathogenesis of severe SFTS. Recent evidence indicates that fatal SFTS is characterized by excessive IL‐6/IL‐10 production accompanied by reduced TGF‐β, reflecting a collapse of immunoregulatory control [27]. Consistent with this pattern, our data show significantly lower TGF‐β1 levels in non‐survivors, suggesting impaired regulatory cytokine activity in fatal cases. Together with our previous findings that SFTS patients exhibit higher TGF‐β1 levels than healthy controls [28], these results imply that inadequate maintenance of TGF‐β1 may contribute to uncontrolled inflammation and poor outcomes. Elevated serum ferritin, often associated with hemophagocytic activity, reflects hyperinflammation and is strongly correlated with poor prognosis in SFTS [6]. Our earlier study also showed that increased PCT levels may indicate secondary bacterial infections, both contributing to higher mortality risk [29]. Collectively, these biomarkers highlight profound immune dysregulation and multiorgan injury, serving as crucial indicators of disease severity and predictors of adverse outcomes in SFTS.

Our findings indicate that B‐cell dysregulation plays a central role in the immunopathogenesis of severe SFTSV infection. In the deceased group, we observed a significant reduction in naïve B‐cells, along with a marked expansion of DNBs, an atypical subset previously associated with infection and autoimmune diseases [13]. DNBs are a heterogeneous subset that may contribute to both pathogen defense and immune dysregulation. Certain subtypes, such as DN2 cells, are associated with differentiation into antibody‐secreting cells under inflammatory conditions [30]. Our results suggest that although plasmablasts expanded in both groups during the acute phase of infection, their levels did not differ significantly. ScRNA‐seq has revealed that plasmablasts harbor the highest viral load in SFTS patients, and their expansion is closely associated with elevated inflammatory cytokines [17, 31]. These shifts in B‐cell profiles suggest an impaired humoral immune response that may underlie the adverse clinical outcomes observed in SFTS [32]. Notably, although plasmablast expansion has been associated with effective viral clearance in infections such as Ebola and other viral diseases [33], our findings indicate that in SFTS, this expansion may not equate to functional antibody production, particularly in fatal cases. Defective virus‐specific humoral immunity represents a key immunopathological feature contributing to mortality in SFTS.

Our integrated prognostic model identified age, viral load, and DNB percentage as independent predictors of 28‐day mortality in SFTS. Advanced age likely reflects immunosenescence and reduced antiviral resilience, while elevated viral load indicates uncontrolled viral replication and immune escape [34, 35]. The expansion of DNBs, a dysfunctional B‐cell subset linked to impaired antibody production and immune exhaustion, highlights the role of humoral immune dysregulation in disease progression. However, the functional characteristics of B‐cell subsets, particularly DNBs, were not fully characterized, and their precise contribution to antibody deficiency and clinical deterioration remains to be elucidated. These prognostic indicators underscore the complex interplay between host immune status, viral dynamics, and immunopathology in shaping SFTS outcomes. Future multicenter studies with larger cohorts, longitudinal sampling, and in‐depth functional analyses are needed to validate these findings and advance our understanding of SFTSV immunopathogenesis.

## 5. Conclusion

This study evaluated B‐cell subpopulations alongside laboratory indicators during the acute phase of SFTSV infection, identifying age, viral load, and DNB percentage as independent risk factors associated with poor prognosis. These findings enhance our understanding of SFTS pathogenesis and support early risk stratification to guide timely interventions and reduce mortality.

## Ethics Statement

This study was approved by the ethical committee of Tongji Hospital, Tongji Medical College, Huazhong University of Science and Technology (TJ‐IRB20230632).

## Conflicts of Interest

The authors declare no conflicts of interest.

## Author Contributions

Hongyan Hou and Heng Liu spearheaded the study’s design, with Qian Tai, Yun Wang, and Renren Ouyang responsible for data collection. Rujia Chen, Ting Wang, and Wei Wei conducted the data analysis, and Hongyan Hou drafted the manuscript. Shiji Wu and Feng Wang provided critical guidance and oversight throughout the research. All authors reviewed and approved the final version of the manuscript. Wei Wei and Qian Tai have contributed equally to this article.

## Funding

This study was funded by the project of the National Natural Science Foundation of China (Number: 82,502,807) and National Key R&D Program of China (grant number 2022YFA1303500).

## Supporting Information

Additional supporting information can be found online in the Supporting Information section.

## Supporting information


**Supporting Information** Figure S1 Gating strategy for B‐cell subsets analysis by flow cytometry. Peripheral blood B‐cells were first gated on singlet, CD45+CD19+ lymphocytes. Plasmablasts were identified as CD27+CD38high cells within the CD19+ population. The remaining nonplasmablast (NPB) B‐cells were further classified into four subsets based on IgD and CD27 expression: naïve B‐cells (IgD+CD27‐), unswitched memory B‐cells (IgD+CD27+), switched memory B‐cells (IgD‐CD27+), and double‐negative B‐cells (IgD‐CD27‐).

## Data Availability

The data that support the findings of this study are available from the corresponding author upon reasonable request.
