# Supplementary figures and images for: Integration of SFTSV Viral Load, Age, and Double‐Negative B‐Cells as Prognostic Biomarkers for Severe Fever With Thrombocytopenia Syndrome Outcomes
Source: J Immunol Res. 2026 Jan 15;2026:8554086. doi: 10.1155/jimr/8554086 (PMC13140936; doi:10.1155/jimr/8554086)

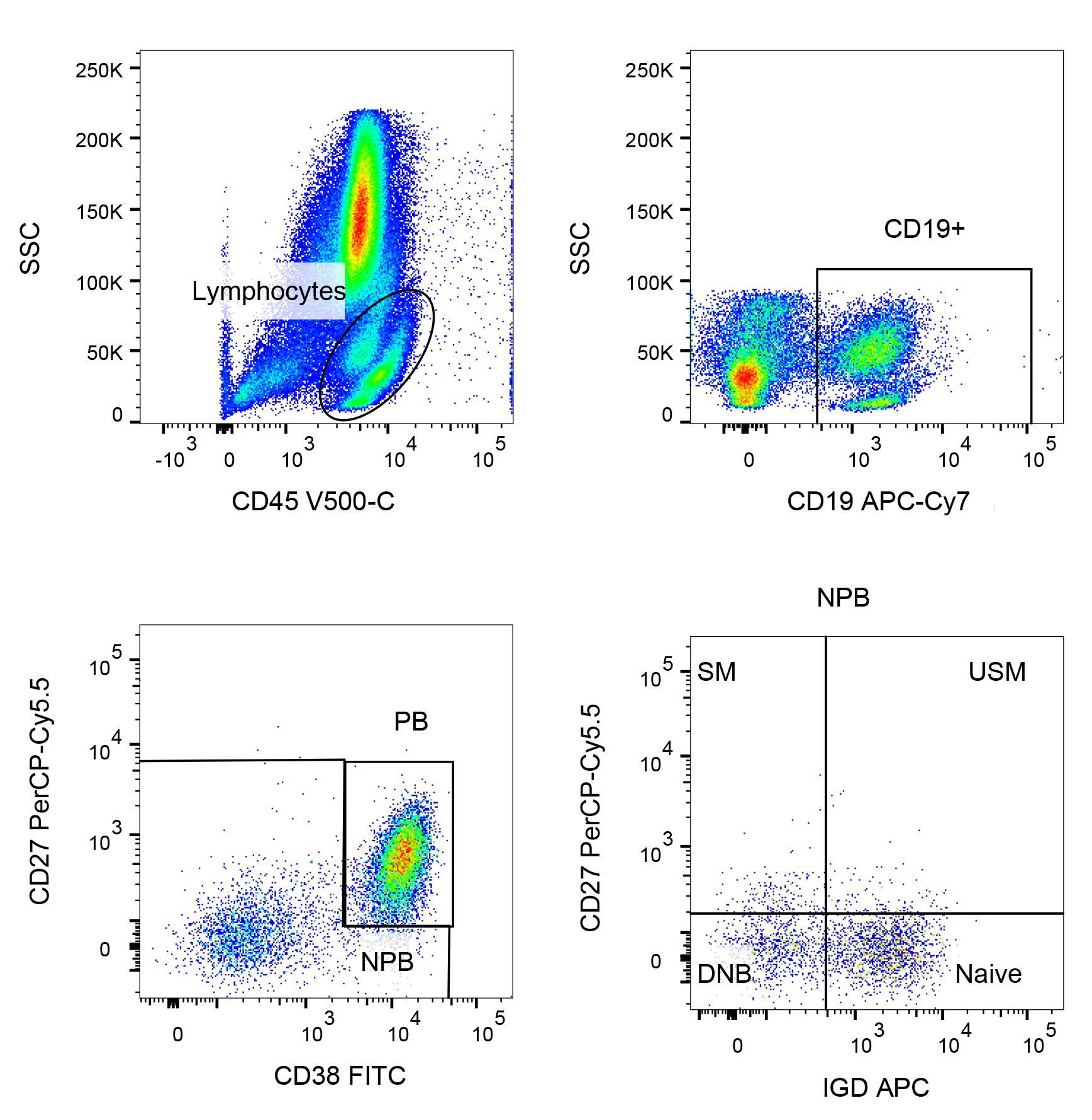

Supplement: Supplementary file 1 — Supporting Information Figure S1 Gating strategy for B‐cell subsets analysis by flow cytometry. Peripheral blood B‐cells were first gated on singlet, CD45+CD19+ lymphocytes. Plasmablasts were identified as CD27+CD38high cells within the CD19+ population. The remaining nonplasmablast (NPB) B‐cells were further classified into four subsets based on IgD and CD27 expression: naïve B‐cells (IgD+CD27‐), unswitched memory B‐cells (IgD+CD27+), switched memory B‐cells (IgD‐CD27+), and double‐negative B‐cells (IgD‐CD27‐). [file JIMR-2026-8554086-s001.jpg]
